# Selegiline Recovers Synaptic Plasticity in the Medial Prefrontal Cortex and Improves Corresponding Depression-Like Behavior in a Mouse Model of Parkinson’s Disease

**DOI:** 10.3389/fnbeh.2019.00176

**Published:** 2019-08-02

**Authors:** Motoki Okano, Kazue Takahata, Junya Sugimoto, Shizuko Muraoka

**Affiliations:** Department of Scientific Research, Fujimoto Pharmaceutical Corporation, Osaka, Japan

**Keywords:** Parkinson’s disease, non-motor symptoms, depression, synaptic plasticity, selegiline, long-term potentiation, CaMKII, MPTP

## Abstract

In patients with Parkinson’s disease (PD), non-motor symptoms (NMS) including depression and anxiety are often recognized before motor symptoms develop. Monoamine oxidase (MAO)-B inhibitors are therapeutically effective for motor symptoms; however, their effects on NMS in PD are yet to be fully assessed. Here, we aimed to explore the antidepressant-like effects of propargyl MAO-B inhibitors, selegiline and rasagiline, in mice treated with 1-methyl-4-phenyl-1,2,3,6-tetrahydropyridine (MPTP) as a PD model, and to elucidate the mechanisms underlying these effects. Four repeated intraperitoneal injections of MPTP at 17.5 mg/kg to C57BL/6 mice led to a partial reduction in the number of nigrostriatal tyrosine hydroxylase-positive neurons and to the extension of immobility time during the tail suspension test (TST), without any obvious induction of motor deficits. A single subcutaneous administration of selegiline at 10 mg/kg shortened the extended immobility time of MPTP mice in the TST, without any increase in motor activities, suggesting that selegiline exerts antidepressant-like effects. In this test, rasagiline did not produce antidepressant-like effects, although the inhibitory effect of 3 mg/kg rasagiline on brain MAO activity was comparable to that of 10 mg/kg selegiline. The shortened immobility time in the TST correlated with reduced cortical dopamine (DA) turnover rates in MPTP mice treated with selegiline, but not in MPTP mice treated with rasagiline. These results suggest that MAO inhibition does not entirely account for the antidepressant-like effects of selegiline. Administration of selegiline (10 mg/kg), but not rasagiline (1 mg/kg), to MPTP mice restored the impaired long-term potentiation induced by high-frequency stimulation in the medial prefrontal cortex (mPFC), and normalized the reduced phosphorylation of Ca^2+^/calmodulin-dependent protein kinase IIα, which is known to be involved in neuroplasticity, in the frontal cortex. In MPTP mice, the antiparkinsonian drug pramipexole (0.3 mg/kg), a DA D_2_ and D_3_ receptor agonist, that has been shown to be effective in treating depression in PD, ameliorated depression-like behavior and synaptic dysfunction in the mPFC. Taken together, the antidepressant-like effects of selegiline in MPTP mice are attributable to the restoration of impaired synaptic plasticity in the mPFC, suggesting its potential for treating depression in early PD.

## Introduction

Parkinson’s disease (PD) is a progressive neurodegenerative disorder characterized by motor symptoms, such as tremor, rigidity, bradykinesia, and postural instability. These symptoms mainly arise from the degeneration of nigrostriatal dopaminergic (DAergic) neurons (Lang and Lozano, 1998). However, non-motor symptoms (NMS) are often present prior to motor symptoms, and they negatively affect the quality of life of patients with PD (Barone et al., 2009). NMS include depression, anxiety, mild cognitive impairment, olfactory dysfunction, and sleep disturbance. Braak et al. (2003) hypothesized that NMS in PD could occur because of degeneration of DAergic and non-DAergic systems in multiple brain regions outside the nigrostriatal DAergic pathway. In addition, NMS have been reported to be unresolvable by dopamine (DA) replacement therapies (Lee and Koh, 2015). Thus, the treatment strategies for NMS need to be differentiated from those for motor symptoms. The results of some clinical studies suggest that antidepressants acting on serotonergic and/or norepinephrinergic systems ameliorate depression in PD (Devos et al., 2008; Menza et al., 2009). Nevertheless, the efficacy of conventional antidepressants for depression in PD is often limited, and some antidepressants are likely to worsen motor symptoms (van de Vijver et al., 2002). Moreover, caution should be exercised when prescribing antidepressants, because some antidepressants may have adverse drug interactions with anti-PD agents (Richard et al., 1997; Hébant et al., 2016). Therefore, a monotherapeutic agent that improves both motor symptoms and NMS would be more desirable than combination treatment with an anti-PD agent and a psychiatric drug; the latter being prescribed sometimes for off-label use.

Selegiline, a selective and irreversible monoamine oxidase (MAO; EC 1.4.3.4)-B inhibitor (MAOBI), has been widely used for PD treatment. In addition, selegiline alleviates clinical symptoms in patients with major depressive disorder (MDD) (Mann et al., 1989), and its transdermal patch is approved for the treatment of MDD in the United States. However, to our knowledge, no clinical trials have been conducted to evaluate the effects of selegiline primarily on psychiatric symptoms in patients with PD. Therefore, we investigated the potential of selegiline in treating depression in PD by using a mouse model of PD. Rasagiline, another MAOBI that also has a propargylamine moiety, was reported to ameliorate depression in PD partly (Barone et al., 2015). Pramipexole and rotigotine, DA D_2_ and D_3_ receptor agonists, have been shown to be effective in treating psychiatric symptoms in PD (Barone et al., 2010; Antonini et al., 2015). However, whether DAergic anti-PD medications and MAOBIs exert similar antidepressant effects in depressed patients with PD and whether the antidepressant effects are independent of their effects on motor symptoms remain to be investigated.

The major cause of PD is degeneration of DAergic neurons that leads to a change in synaptic plasticity in the corticostriatal pathway, followed by the onset of motor symptoms (Calabresi et al., 2009; Udupa and Chen, 2013; Zhuang et al., 2013). The pathology underlying psychiatric symptoms in PD has been reported to involve regions other than motor loops, such as the prefrontal cortex (PFC) (Andersen et al., 2015; Gatt et al., 2016), entorhinal cortex (Goldman et al., 2012), and hippocampus (van Mierlo et al., 2015; Yildiz et al., 2015; Györfi et al., 2017; Gou et al., 2018). Depressed patients with PD fail to suppress activities in the PFC of the default-mode network (Andersen et al., 2015). It is unclear whether depressed patients with PD show any impairment in long-term potentiation (LTP)-like plasticity in the PFC; patients with MDD do show such impairment (Normann et al., 2007; Kuhn et al., 2016). The impairment in high-frequency stimulation (HFS)-induced LTP in the hippocampus–PFC pathway of rats exposed to stress, a key trigger for onset of MDD, can be reversed under the influence of some antidepressants (Rocher et al., 2004). To our knowledge, there have been no reports showing the relationship between cortical LTP impairment and depression-like behavior in PD animal models, although it has been reported that HFS-induced LTP in the hippocampus is impaired in PD animal models having cognitive deficits (Costa et al., 2012; Diógenes et al., 2012; Moriguchi et al., 2012; Bonito-Oliva et al., 2014b; Castro-Hernández et al., 2017).

Considering the above issues with respect to the potential of MAOBIs, we assessed their effects on NMS-like behavior in 1-methyl-4-phenyl-1,2,3,6-tetrahydropyridine (MPTP)-treated C57BL/6 mice (MPTP mice), which are widely used as a PD animal model exhibiting NMS-like behavior as well as motor impairment (Mori et al., 2005; Rojas et al., 2008; Hutter-Saunders et al., 2011; Moriguchi et al., 2012; Shin et al., 2014). Furthermore, we evaluated the effects of MAOBIs on synaptic plasticity in the hippocampus–PFC pathway in MPTP mice, and on the expression and phosphorylation of Ca^2+^/calmodulin-dependent protein kinase (CaMK; EC: 2.7.11.17) II, which is known to be involved in neuroplasticity. Our studies show significant differences in efficacies between propargyl MAOBIs on depression-like behavior and impaired synaptic plasticity in the medial PFC (mPFC) in MPTP mice, and these results suggest the potential application of selegiline for the treatment of depression in early PD.

## Materials and Methods

### Animals

Male C57BL/6J mice (8–10 weeks old, weighing 20–27 g) were purchased from Charles River Laboratories Japan (RRID:IMSR_JAX:000664; Kanagawa, Japan). Upon arrival, the mice were maintained in the facility with controlled humidity (50 ± 20%) and temperature (23 ± 3°C), under a 12-h light/dark cycle (light on at 7:00 a.m.), with free access to food (MF chow pellets, Oriental Yeast, Tokyo, Japan) and water. The mice were housed in plastic cages (182 × 260 × 128 mm, CLEA Japan Inc., Tokyo, Japan) with sterilized paper bedding (Paperclean, Nihon SLC, Shizuoka, Japan), and acclimated for at least 4 days prior to experiments. The mice were identified by tail marking and grouped based on computer-based randomization by body weight stratification. All animal procedures were carried out in accordance with the applicable international, national, and institutional guidelines for the care and use of animals. All experiments were approved by the Committee of Fujimoto Pharmaceutical Corporation on Animal Experimentation (the approval number: AC-F-2666).

### Drugs

Selegiline hydrochloride (0.3, 1, 3, and 10 mg/kg; Fujimoto Pharmaceutical Corp., Osaka, Japan), rasagiline mesylate (0.1, 0.3, 1, and 3 mg/kg; Sigma-Aldrich, St. Louis, MO, United States; catalog number: SML0124), and pramipexole dihydrochloride monohydrate (0.1, 0.3, and 1 mg/kg; Wako Pure Chemical Industries, Osaka, Japan; catalog number: 169-26183) were dissolved in saline and administered subcutaneously (s.c.) 60 min before behavioral tests or HFS in the electrophysiological study. Benserazide hydrochloride (Sigma-Aldrich, St. Louis, MO, United States; catalog number: B7283) was dissolved in saline and administered intraperitoneally (i.p.) at a dose of 2.5 or 6.25 mg/kg 15 min before injection of 3,4-dihydroxy-L-phenylalanine (L-Dopa; Sigma-Aldrich, St. Louis, MO, United States; catalog number: D9628). L-Dopa was suspended in saline containing 0.25% (w/v) carboxymethylcellulose sodium and administered i.p. at a dose of 10 or 25 mg/kg 30 min before measurement of motor functions. All compounds were administered to mice in a volume of 10 mL/kg.

### MPTP Treatment

**FIGURE 1:**
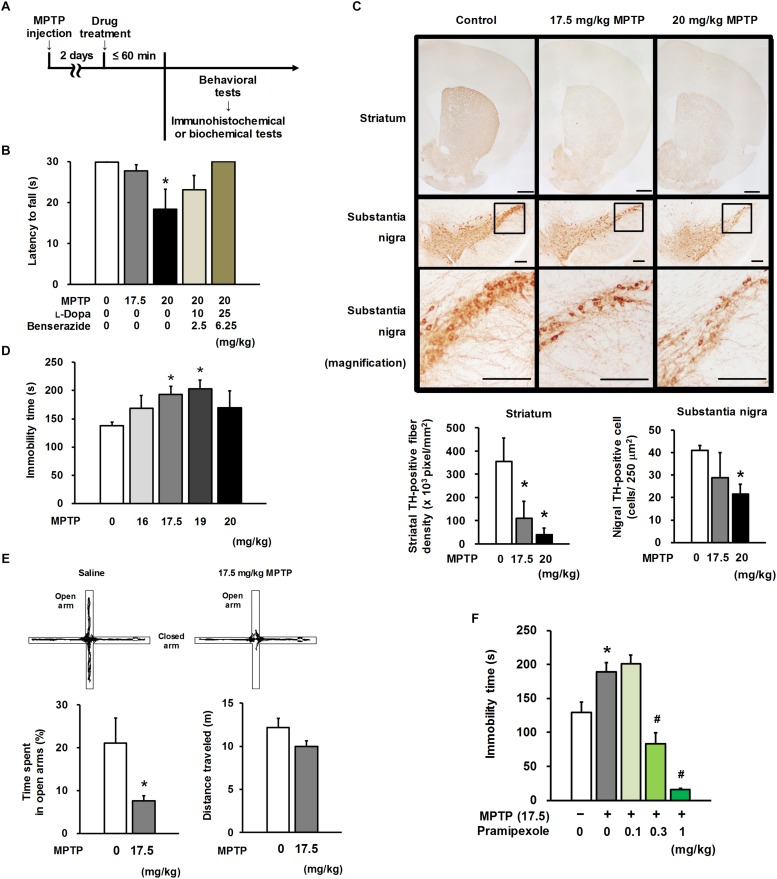
Motor and non-motor characteristics in MPTP mice. **(A)** Experimental timeline. **(B)** Four repeated intraperitoneal injections of 1-methyl-4-phenyl-1,2,3,6-tetrahydropyridine (MPTP) at 20 mg/kg, but not 17.5 mg/kg, caused motor dysfunction in the horizontal bar test. Combined treatment with L-Dopa and benserazide (30 and 45 min before the behavioral test, respectively) ameliorated MPTP-induced motor dysfunction. The groups were control (*n* = 13), 17.5 mg/kg MPTP (*n* = 12), 20 mg/kg MPTP (*n* = 5), 20 mg/kg MPTP + 10 mg/kg L-Dopa (*n* = 5), and 20 mg/kg MPTP + 25 mg/kg L-Dopa (*n* = 5). Values represent means ± SEM. ^*^p < 0.05 versus the control group (Dunn’s test using Bonferroni adjustment). **(C)** Representative photomicrographs of tyrosine hydroxylase (TH) immunohistochemistry in the striatum and substantia nigra of MPTP- or saline-treated mice (upper). Administration of MPTP significantly induced a reduction in TH-positive striatal nerve terminals (lower left) and TH-positive nigral cells (lower right). Scale bar = 500 μm (striatum) and 200 μm (substantia nigra). The groups were control (*n* = 3–4), 17.5 mg/kg MPTP (*n* = 4), and 20 mg/kg MPTP (*n* = 3–4). Values represent means ± standard deviation (SD). ^*^p < 0.05 versus the control group (Dunnett’s test), striatum: *F*_(2,9)_ = 20.325, *p* < 0.05; substantia nigra: *F*_(2,7)_ = 5.040, *p* < 0.05. **(D)** MPTP mice exhibited depression-like behavior in the tail suspension test (TST). The groups were control (*n* = 11), 16 mg/kg MPTP (*n* = 5), 17.5 mg/kg MPTP (*n* = 5), 19 mg/kg MPTP (*n* = 4), and 20 mg/kg MPTP (*n* = 4). Values represent means ± SEM. ^*^p < 0.05 versus the control group (Dunnett’s test), *F*_(4,24)_ = 3.146, *p* < 0.05. **(E)** Representative traces of MPTP- or saline-treated mice in the elevated plus maze (EPM) test (upper). Anxiety-like behavior (lower left) and distance traveled (lower right) in the EPM test in MPTP mice. The groups were control (*n* = 11) and 17.5 mg/kg MPTP (*n* = 10). Values represent means ± SEM. ^*^p < 0.05 versus the control group (Welch’s *t*-test). **(F)** Pramipexole (PRX) was administered 60 min before the TST. PRX improved depression-like behavior in the TST in MPTP mice. The groups were control (*n =* 7), 17.5 mg/kg MPTP + saline (*n =* 6), 17.5 mg/kg MPTP + 0.1 mg/kg PRX (*n =* 4), 17.5 mg/kg MPTP + 0.3 mg/kg PRX (*n =* 4), and 17.5 mg/kg MPTP + 1 mg/kg PRX (*n =* 3). Values represent means ± SD. ^*^p < 0.05 versus the control group, ^#^*p* < 0.05 versus the MPTP + saline group (Tukey’s test), *F*_(4, 19)_ = 20.906, *p* < 0.05.

1-Methyl-4-phenyl-1,2,3,6-tetrahydropyridine hydrochloride (Sigma-Aldrich, St. Louis, MO, United States; catalog number: M0896; two independent batches) was dissolved in saline. The mice received four i.p. injections of saline or MPTP at a dose of 16, 17.5, 19, or 20 mg free base/kg at 2-h intervals, according to the method described previously by Mori et al. (2005). The mice were kept warm at 25 ± 1°C in metal cages (170 × 300 × 100 mm, Natsume Seisakusho Co., Tokyo, Japan) until the morning after the third MPTP injection to prevent fatal hypothermia, according to the method described previously by Jiao et al. (2015). In the present study, 20 of 513 mice died owing to unexpected adverse events, probably cardiac abnormalities after administration of MPTP. All experiments were performed 2 days after MPTP treatment (Figures 1A, 4A). Behavioral tests were performed with two independent batches of mice.

### Motor Function

Locomotor activities of each mouse were automatically measured by an infrared-linked activity sensor (Neuroscience, Tokyo, Japan) at 5 min intervals for 30 min in a plastic cage (13 × 18 × 26 cm). The horizontal bar test (HBT) was performed according to the method described by Deacon (2013). Briefly, the mice were allowed to grasp the center of a horizontal wooden bar (diameter: 0.6 cm, width: 38 cm; 20 cm above the bench surface) with their forelimbs. Then, the latency to fall from the bar was manually measured within 30 s by an observer blinded to the treatment conditions.

### Depression-Like Behavior

Depression-like behavior was assessed by the tail suspension test (TST), according to the methods described by Steru et al. (1985) and Can et al. (2012). Briefly, each mouse was suspended from a bar by the tail with an adhesive tape in a three-walled compartment (55 × 15 × 15 cm). The mouse tail base area was covered with a transparent plastic tube (3 cm length) to prevent tail climbing behavior. The duration of immobility (defined as the time spent without any limb movement) was manually measured for 6 min by an observer blinded to the treatment conditions.

### Anxiety-Like Behavior

Anxiety-like behavior was assessed by the elevated plus maze (EPM) test, according to the method described by Lister (1987). Briefly, the mice were placed on the intersection (neutral zone) of an opaque white plastic plus maze (four arms: width: 4 cm, length: 25 cm; height: 50 cm). Two opposing arms were enclosed by transparent plastic walls (height: 10 cm), and the two open arms had transparent edges (height: 0.3 cm). The time spent in the open arms and the distance traveled were measured for 10 min by the tracking software EthoVision 3.0.13 (Noldus, Wageningen, Netherlands). The treatment conditions were not blinded.

### Tyrosine Hydroxylase Immunoreactivity

Immediately after the HBT, the mice were sacrificed by cervical dislocation. After decapitation, their brains were dissected, fixed in 10% (v/v) neutral buffered formalin for 2 days, and embedded in paraffin. Coronal sections (thickness: 20 μm), including the substantia nigra (ranging from 2.8 to 3.2 mm posterior to the bregma) or the striatum (ranging from 1.5 to 0.7 mm anterior to the bregma), were cut with a microtome, deparaffinized with xylene, and subjected to antigen retrieval with 0.1% (v/v) trypsin. The sections were incubated in 0.3% (v/v) hydrogen peroxide in methanol, then in a blocking buffer [5% (v/v) normal goat serum in phosphate-buffered saline containing 0.2% (v/v) Triton-X] for 30 min, and further incubated at 4°C with a rabbit polyclonal antibody against tyrosine hydroxylase (TH; EC 1.14.16.2) diluted in the blocking buffer (RRID:AB_390204; 1:500, Millipore, Billerica, MA, United States; Shin et al., 2014). After incubation with biotinylated goat anti-rabbit IgG antibody in blocking buffer (RRID:AB_2313606; 1:500, Vector, Burlingame, CA, United States), the sections were incubated with an avidin-biotinylated horseradish peroxidase (HRP; EC 1.11.1.7) complex using VECTASTAIN ABC Standard Kit (RRID:AB_2336818; Vector, Burlingame, CA, United States). TH immunoreactivity was visualized by the 3,3′-diaminobenzidine staining method. TH-positive cells in the substantia nigra were counted inside a 500 × 500 μm counting frame by an observer blinded to the treatment conditions. The striatal TH-positive fiber density was analyzed in a 500 × 500 μm frame of the dorsolateral striatum by using the image analysis software WinROOF 7.0.0 (Mitani, Tokyo, Japan). The optical density in the dorsolateral striatum was corrected for non-specific background staining in the cerebral cortex by subtracting such staining.

### MAO Activities

Immediately after the TST or EPM, the mice were sacrificed by cervical dislocation. After decapitation, the cerebrum was dissected and stored at −80°C until measurement. MAO-A and -B activities in the cerebral mitochondria were measured by using serotonin (5-HT) and benzylamine, respectively, as substrates, together with the EnzyChrom MAO Assay Kit (BioAssay Systems, Hayward, CA, United States), according to the manufacturer’s instructions with a slight modification (substrate alteration).

### Content of Monoamines and Their Metabolites

The mice subjected to the TST were sacrificed by cervical dislocation, and their brains were rapidly removed after decapitation. The striatum and cerebral cortex were dissected and stored at −80°C until sample preparation. The sample preparation was carried out according to the method previously described by Kasai et al. (2017). Briefly, tissues were homogenized in 0.2 M perchloric acid containing isoproterenol (for striatum: 100 pg/mL; cerebral cortex: 10 pg/μL) as an internal standard. The homogenates were kept on ice for 30 min and centrifuged for 20 min at 15,000 × *g* at 4°C. The supernatants were passed through a 0.45-μm filter membrane. The filtered supernatants were stored at −80°C until high-performance liquid chromatography (HPLC) measurement. The tissue content of DA and its metabolites, 3,4-dihydroxyphenylacetic acid (DOPAC) and homovanillic acid (HVA), was measured in a HPLC-electrochemical detection system (ECD-700, Eicom Corp., Kyoto, Japan). 5-HT and its metabolite 5-hydroxyindoleacetic acid, and norepinephrine (NA) and its metabolite 3-methoxy-4-hydroxyphenylglycol, were also measured. Each sample was injected into a C18 reverse-phase column (Eicompak SC-5ODS: 3.0 mm × 150 mm, Eicom Corp., Kyoto, Japan) conditioned at 25°C. The mobile phase comprising 0.1 M acetic acid–citric acid buffer (pH 3.5), 15% (v/v) methanol, 190 mg/L sodium 1-octanesulfonate, and 5 mg/L ethylenediaminetetraacetic acid was delivered at a flow rate of 0.5 mL/min. The applied potential was set at +750 mV versus Ag/AgCl. The content of monoamines and their metabolites was calculated using standard curves, and expressed as μg/g wet tissue.

### Electrophysiological Experiments for Assessment of Synaptic Plasticity

Electrophysiological tests were performed by using the method described previously by Izaki et al. (2001) and Hiraide et al. (2012). Under urethane anesthesia (1.5 g/kg, i.p.), a bipolar stimulating electrode was stereotaxically placed in the CA1/subicular region of the ventral hippocampus [Anterior–Posterior (AP): −3.6 mm, Medial–Lateral (ML): +3.4 mm, Dorsal–Ventral (DV): −2.0 to −3.5 mm from the bregma], and a recording electrode (stainless steel, 100-μm diameter) was lowered into the mPFC (AP: +1.9 mm, ML: +0.4 mm, DV: −1.3 to −1.8 mm from the bregma) according to the mouse brain atlas (Franklin and Paxinos, 2008). The population spike amplitude (PSA) in the mPFC was obtained from seven stimuli at 30-s intervals, and was recorded every 5 min by the data analysis software Scope 3.7.6 (AD Instruments, Sydney, Australia). The intensity of stimulation was adjusted for each mouse to elicit a PSA of approximately 60% of maximum amplitude (pulse duration: 250 μs). The PSA was expressed as a percentage of the baseline value before application of two series of HFS (10 stimuli/train at 10-s intertrain intervals, 50 trains at 4-ms intervals) at a 10 min interval. Area under the curve (AUC) of PSA for 60 min after the first HFS was calculated. The treatment conditions were not blinded.

### Western Blot Analysis of Total and Phosphorylated CaMKII Levels

One hour after a single injection of the drugs, mice were sacrificed by cervical dislocation and decapitated. The frontal cortices were dissected by using a mouse brain slicer matrix and stored at −80°C until use. Tissues were homogenized in radio-immunoprecipitation assay buffer comprising 25 mM Tris–HCl (pH 7.6), 150 mM NaCl, 1% (v/v) NP-40, 1% (w/v) sodium deoxycholate, 0.1% (w/v) sodium dodecyl sulfate (SDS), 1× phosphatase/protease inhibitor cocktail (Thermo Scientific, Waltham, MA, United States), and 1 mM phenylmethylsulfonyl fluoride. After centrifugation at 1,000 × *g* for 10 min, the supernatants were collected and centrifuged at 15,000 × *g* for 20 min at 4°C. The resultant supernatants were collected, and their total protein concentrations were determined by the Lowry method (DC Protein Assay, Bio-Rad, Hercules, CA, United States). The samples were denatured by boiling for 5 min in Laemmli sample buffer. Ten micrograms of protein was separated by electrophoresis through 9% (w/v) SDS–polyacrylamide gel and transferred to polyvinylidene difluoride membranes (Millipore, Billerica, MA, United States). The membranes were blocked with 5% (w/v) skim milk in Tris-buffered saline containing 0.05% (w/v) Tween 20, and then incubated with the following primary antibodies: a polyclonal antibody against rabbit CaMKII (RRID:AB_2067912; 1:5,000, Santa Cruz, Dallas, TX, United States) or against threonine-286 phosphorylated-CaMKII (RRID:AB_310282; 1:1,000, Millipore, Billerica, MA, United States; Anderson et al., 2008) and a monoclonal antibody against mouse β-actin (RRID:AB_476697; 1:10,000, Sigma-Aldrich, St. Louis, MO, United States). After incubation with HRP-conjugated secondary antibodies (RRID:AB_228341 or AB_228427), immunoreactive signals were detected by using Luminata Forte Western HRP Substrate (Millipore, Billerica, MA, United States), and the band intensities were quantified using the image analysis software ImageLab 3.0 (Bio-Rad, Hercules, CA, United States).

### Statistical Analysis

Statistical analyses were performed with SPSS 23.0 (IBM Corp., Armonk, NY, United States). Welch’s *t*-test was used to evaluate the difference between two groups with unequal variance. One-way ANOVA was used to compare more than two groups, followed by *post hoc* Dunnett’s, Tukey’s (equal variance), or Games–Howell (unequal variance) tests. Non-parametric data were analyzed by the Kruskal–Wallis test, followed by a *post hoc* Dunn’s test with Bonferroni adjustment. Possible correlation between immobility time in the TST and monoamine content or its turnover rate was analyzed by Pearson’s correlation test or Spearman’s non-parametric rank correlation test. Difference and correlation were considered significant at *p* < 0.05. In the present study, 20 of 493 animals were excluded before conducting analyses owing to incomplete testing due to technical insufficiency. The exact numbers for all experiments are presented in the figure legends.

## Results

### Motor and Non-motor Characteristics of MPTP Mice

Four repeated i.p. injections of MPTP to C57BL/6J mice at 20 mg/kg, but not 17.5 mg/kg, resulted in a significant reduction in suspension time in the HBT (20 mg/kg, *p* = 0.026). This motor impairment in 20 mg/kg-MPTP mice was ameliorated in a dose-dependent manner through combined treatment with L-Dopa and benserazide (Figure 1B). Administration of MPTP at a dose of 17.5 or 20 mg/kg significantly reduced the TH-positive fiber density in the striatum (17.5 mg/kg, *p* = 0.002; 20 mg/kg, *p* < 0.001). MPTP at 20 mg/kg, but not 17.5 mg/kg, induced a reduction in the number of TH-positive nigral cells (20 mg/kg, *p* = 0.029; Figure 1C). The mice that received MPTP at a dose of 17.5 or 19 mg/kg showed significantly longer immobility time than saline-treated mice (control mice) in the TST, a standard behavioral paradigm indicative of depression (17.5 mg/kg, *p* = 0.046; 19 mg/kg, *p* = 0.026; Figure 1D). In the 17.5 mg/kg-MPTP mice, a significant reduction in time length spent in the open arms of the EPM, a standard behavioral paradigm indicative of anxiety (*p* = 0.043; Figure 1E), was observed; however, the reduction did not correlate with the distance traveled (*r* = 0.383, *p* = 0.086). Although 17.5 mg/kg-MPTP treatment did not induce any apparent motor impairment, as shown in the results of the HBT (Figure 1B) and EPM (Figure 1E, distance traveled), it led to the extension of immobility time in the TST (Figure 1D) and to the reduction in time spent in the open arms of the EPM, suggesting that the 17.5 mg/kg-MPTP mice exhibited depression- and anxiety-like behavior. Thus, we chose 17.5 mg/kg MPTP for preparation of a premotor PD model in the subsequent experiments.

### Effects of the DA Agonist, Pramipexole, on Depression-Like Behavior in MPTP Mice

An anti-PD drug, pramipexole (a DA D_2_ and D_3_ receptor agonist), has been reported to be effective in treating depression associated with PD in randomized clinical trials (Barone et al., 2010; Seppi et al., 2011). Thus, to confirm the validity of the present MPTP-treated animal model for mimicking psychiatric symptoms in PD and to investigate any involvement of DAergic actions in depression-like behavior, pramipexole was administered to MPTP mice as a reference drug. A single s.c. administration of pramipexole shortened the extended immobility time of MPTP mice in the TST (0.3 mg/kg, *p* = 0.001; 1 mg/kg, *p* < 0.001; Figure 1F) in a dose-dependent manner, while 0.1–1 mg/kg doses did not increase spontaneous locomotor activities (Supplementary Figure 1). These findings indicate that pramipexole alleviates depression-like behavior in MPTP mice. For the next set of experiments, pramipexole at a dose of 0.3 mg/kg, which had shown a substantial recovery potential for MPTP-induced extended immobility time, was selected to compare its effects on NMS-like behavior with those of MAOBIs.

### Effects of MAOBIs on Depression- and Anxiety-Like Behavior in MPTP Mice

**FIGURE 2:**
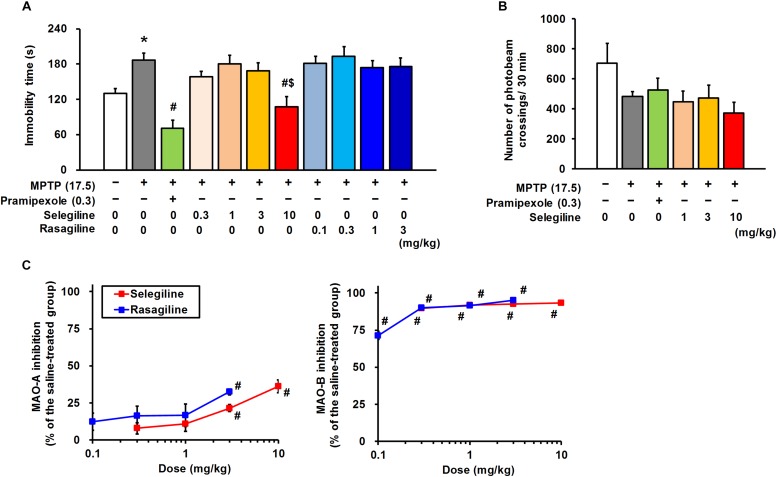
Effects of monoamine oxidase-B inhibitors, selegiline (SEL) and rasagiline (RAS), and pramipexole (PRX) on depression-like behavior in MPTP mice. **(A)** Administration of SEL or PRX, but not RAS, ameliorated the depression-like behavior of MPTP mice. The groups were control (*n* = 11), MPTP + saline (*n* = 11), MPTP + PRX (*n* = 8), MPTP + 0.3 mg/kg SEL (*n* = 7), MPTP + 1 mg/kg SEL (*n* = 7), MPTP + 3 mg/kg SEL (*n* = 7), MPTP + 10 mg/kg SEL (*n* = 8), MPTP + 0.1 mg/kg RAS (*n* = 7), MPTP + 0.3 mg/kg RAS (*n =* 7), MPTP + 1 mg/kg RAS (*n =* 8), and MPTP + 3 mg/kg RAS (*n =* 7). Values represent means ± SEM. ^*^p < 0.05 versus the control group, ^#^*p* < 0.05 versus the MPTP + saline group, ^$^*p* < 0.05 versus the MPTP + 1 or 3 mg/kg RAS group (Tukey’s test), *F*_(10,77)_ = 8.463, *p* < 0.05. **(B)** Neither SEL nor PRX influenced spontaneous locomotor activity in MPTP mice. The groups were control (*n =* 7), MPTP + saline (*n =* 7), MPTP + PRX (*n =* 7), MPTP + 1 mg/kg SEL (*n =* 7), MPTP + 3 mg/kg SEL (*n =* 7), and MPTP + 10 mg/kg SEL (*n =* 7). Values represent means ± SEM. *F*_(5,36)_ = 1.676, *p* = 0.165. **(C)** Inhibitory effects of SEL and RAS on the activity of MAO-A and -B. The groups were MPTP + saline (*n =* 9), MPTP + 0.3 mg/kg SEL (*n =* 7), MPTP + 1 mg/kg SEL (*n =* 6), MPTP + 3 mg/kg SEL (*n =* 6), MPTP + 10 mg/kg SEL (*n =* 6), MPTP + 0.1 mg/kg RAS (*n =* 6), MPTP + 0.3 mg/kg RAS (*n =* 5), MPTP + 1 mg/kg RAS (*n =* 6), and MPTP + 3 mg/kg RAS (*n =* 7). Values represent means ± SEM. ^#^*p* < 0.05 versus the MPTP + saline group (Games–Howell test), MAO-A: *F*_(8,49)_ = 6.498, *p* < 0.05; MAO-B: *F*_(8,49)_ = 43.101, *p* < 0.05.

When 0.3–10 mg/kg selegiline was administered by a single s.c. injection at 60 min before the TST, the 10 mg/kg dose shortened the extended immobility time of MPTP mice (MPTP versus control mice, *p* = 0.026; 10 mg/kg selegiline-treated versus saline-treated MPTP mice, *p* = 0.001). This recovery effect of 10 mg/kg selegiline on the immobility time of MPTP mice was comparable to that of 0.3 mg/kg pramipexole (pramipexole-treated versus saline-treated MPTP mice, *p* < 0.001; Figure 2A). Similar to pramipexole, selegiline at any dose did not significantly increase the spontaneous locomotor activities in MPTP mice (Figure 2B).

Rasagiline, another MAOBI, has been reported to be 3–15 times more potent than selegiline in terms of MAO-B inhibition in a rodent brain *in vivo* (Youdim et al., 2001). Moreover, the clinical dose of rasagiline is approximately one-tenth of the selegiline dose in patients with PD (Tomlinson et al., 2010), and based on this ratio, pharmacological effects of selegiline and rasagiline in rodents have been compared at doses of 10 and 1 mg/kg, respectively (Abassi et al., 2004; Kasai et al., 2017). In the cerebrum of MPTP mice, selegiline (0.3–10 mg/kg) and rasagiline (0.1–3 mg/kg) markedly and comparably inhibited MAO-B activities (selegiline: 89.8–93.4%, rasagiline: 71.4–95.2%). In addition, selegiline and rasagiline at doses greater than or at 3 mg/kg, respectively, suppressed MAO-A activities significantly (3 or 10 mg/kg selegiline-treated versus saline-treated MPTP mice, *p* = 0.003 or *p* = 0.003; 3 mg/kg rasagiline-treated versus saline-treated MPTP mice, *p* < 0.001). The MAO-A-inhibitory activity of 10 mg/kg selegiline (36.2 ± 4.4%) was comparable to that of 3 mg/kg rasagiline (32.5 ± 1.9%; Figure 2C). Hence, we compared the effects of 0.3–10 mg/kg selegiline with those of 0.1–3 mg/kg rasagiline on depression-like behavior to clarify whether these MAOBIs administered for PD treatment exert comparable antidepressant efficacies. Rasagiline did not show antidepressant-like effects at any dose in MPTP mice. Selegiline at a dose of 10 mg/kg showed more potent efficacy in treating depression-like behavior in MPTP mice than rasagiline (10 mg/kg selegiline versus 1 or 3 mg/kg rasagiline, *p* < 0.05; Figure 2A).

In the EPM test, a single administration of either selegiline (1–10 mg/kg) or rasagiline (0.1–1 mg/kg) did not significantly recover MPTP-induced reduction in time spent in the open arms (MPTP versus control mice, *p* = 0.017) or the distance traveled (MPTP versus control mice, *p* < 0.001; Supplementary Figure 2). These results suggest that a single administration of selegiline has antidepressant-like effects, but not anxiolytic-like effects in MPTP mice, and its effect is neither a consequence of MAO inhibition nor of an increase in motor activities.

### Effects of MAOBIs and Pramipexole on Monoamine Turnover in the Striatum and Cerebral Cortex of MPTP Mice

**FIGURE 3:**
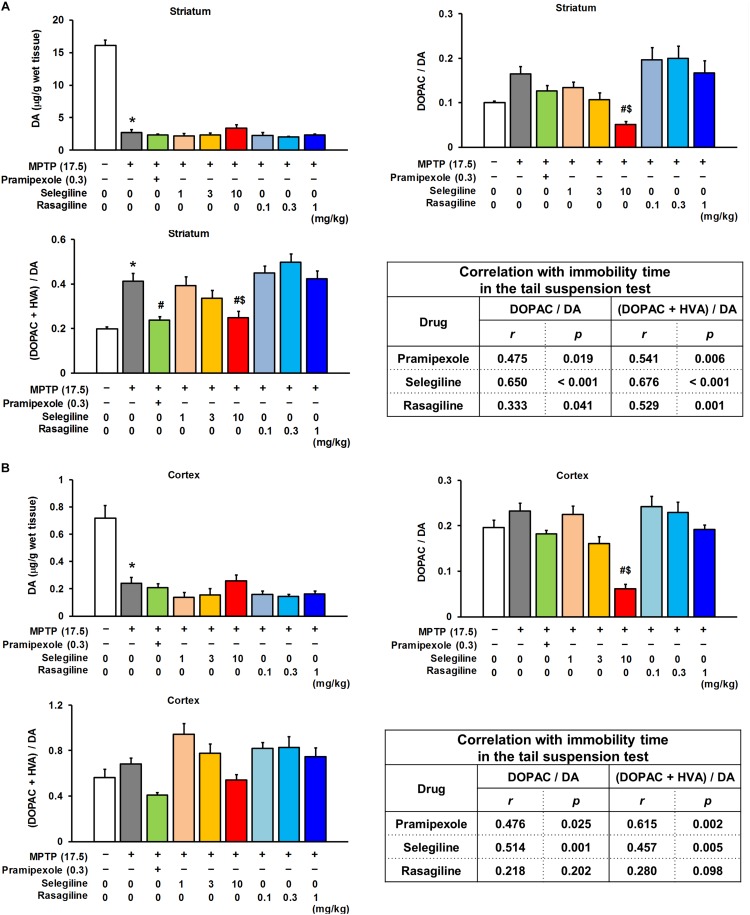
Effects of selegiline (SEL), rasagiline (RAS), and pramipexole (PRX) on dopamine (DA) content and its turnover rate in the striatum and cortex of MPTP mice. **(A)** Effects of SEL, RAS, and PRX on DA content and its turnover rate in the striatum. The groups were control (*n =* 8), MPTP + saline (*n =* 8), MPTP + PRX (*n =* 8), MPTP + 1 mg/kg SEL (*n =* 7), MPTP + 3 mg/kg SEL (*n =* 7), MPTP + 10 mg/kg SEL (*n =* 8), MPTP + 0.1 mg/kg RAS (*n =* 7), MPTP + 0.3 mg/kg RAS (*n =* 7), and MPTP + 1 mg/kg RAS (*n =* 8). Values represent means ± SEM. ^*^p < 0.05 versus the control group. ^#^*p* < 0.05 versus the MPTP + saline group. ^$^*p* < 0.05 versus the MPTP + 1 mg/kg RAS group {DA content and 3,4-dihydroxyphenylacetic acid (DOPAC)/DA: Games–Howell test [DOPAC + homovanillic acid (HVA)]/DA: Tukey’s test}, DA content: *F*_(8,59)_ = 107.131, *p* < 0.05; DOPAC/DA: *F*_(8,59)_ = 6.877, *p* < 0.05; (DOPAC + HVA)/DA: *F*_(8,59)_ = 10.808, *p* < 0.05. **(B)** Effects of SEL, RAS, and PRX on DA content and its turnover rate in the cortex. The groups were control (*n =* 8), MPTP + saline (*n =* 6), MPTP + PRX (*n =* 8), MPTP + 1 mg/kg SEL (*n =* 7), MPTP + 3 mg/kg SEL (*n =* 7), MPTP + 10 mg/kg SEL (*n =* 8), MPTP + 0.1 mg/kg RAS (*n =* 7), MPTP + 0.3 mg/kg RAS (*n =* 7), and MPTP + 1 mg/kg RAS (*n =* 8). Values represent means ± SEM. ^#^*p* < 0.05 versus the MPTP + saline group. ^$^*p* < 0.05 versus the MPTP + 1 mg/kg RAS group (DA content: Games–Howell test; DA turnover: Tukey’s test), DA content: *F*_(8,57)_ = 16.249, *p* < 0.05; DOPAC/DA: *F*_(8,57)_ = 12.061, *p* < 0.05; (DOPAC + HVA)/DA: *F*_(8,57)_ = 5.767, *p* < 0.05.

We next evaluated the effects of MAOBIs and pramipexole on the striatal DA content and its turnover rate, and on the content of cortical DA, 5-HT, and NA, and their turnover rates in MPTP mice subjected to the TST. Selegiline (10 mg/kg) and pramipexole (0.3 mg/kg) significantly lowered the striatal DA turnover rate that had been elevated by MPTP, whereas rasagiline (0.1–1 mg/kg) did not (ratio of DOPAC and HVA to DA: MPTP versus control, *p* < 0.001; pramipexole-treated versus saline-treated MPTP mice, *p* = 0.006; 10 mg/kg selegiline-treated versus saline-treated MPTP mice, *p* = 0.013; Figure 3A). A positive correlation was observed between the striatal DA turnover rate (ratio of DOPAC and HVA to DA) and the immobility time in the TST in each treatment group (selegiline: *r* = 0.676, *p* < 0.001; pramipexole: *r* = 0.541, *p* = 0.006; rasagiline: *r* = 0.529, *p* = 0.001; Figure 3A). MPTP treatment decreased DA and 5-HT content (*p* < 0.05), but did not significantly influence turnover rates of DA, 5-HT, and NA in the cortex (Figure 3B and Supplementary Figure 3).

The cortical DA turnover rate was markedly reduced by selegiline (10 mg/kg), but not by rasagiline or pramipexole (ratio of DOPAC to DA: selegiline-treated versus saline-treated MPTP mice, *p* < 0.001), and positively correlated with the immobility time in the TST in selegiline-treated MPTP mice (*r* = 0.514, *p* = 0.001; Figure 3B). Moderately positive correlations were also observed between the cortical 5-HT or NA turnover rates and the immobility time of mice that had received selegiline (5-HT: *r* = 0.568, *p* < 0.001; NA: *r* = 0.356, *p* = 0.033; Supplementary Figure 3). These results suggest that the antidepressant-like effects of selegiline in MPTP mice are attributable to the normalization of impaired nigrostriatal DAergic systems and to the enhancement of cortical DAergic systems. In addition, these results suggest that the antidepressant-like effect of pramipexole is mediated partly by normalization of the striatal DA turnover.

### Changes in Synaptic Plasticity in the Frontal Cortex of MPTP Mice and Effects of MAOBIs and Pramipexole

**FIGURE 4:**
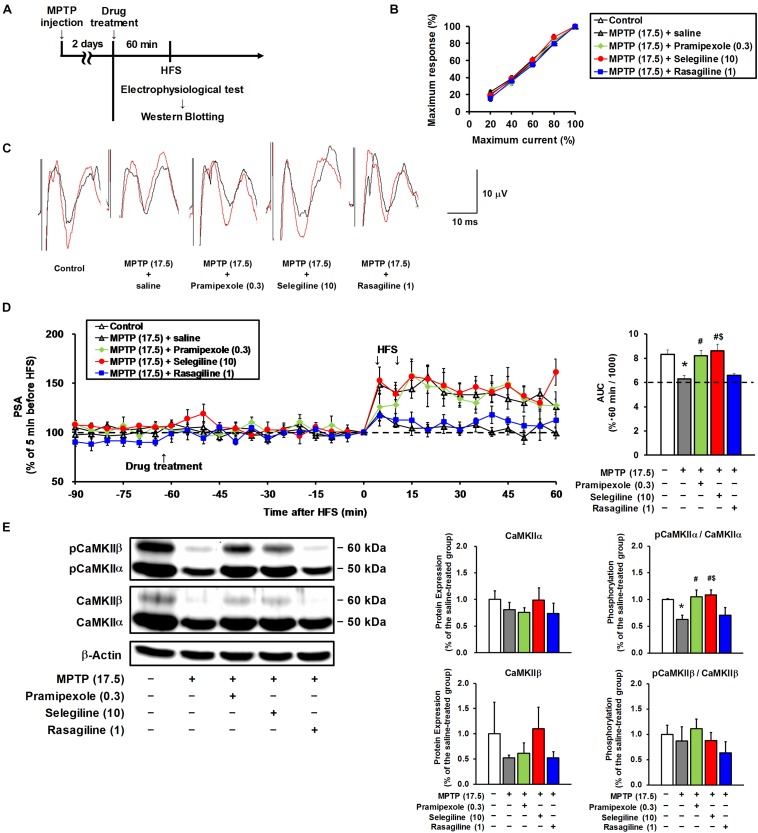
Effects of selegiline (SEL), rasagiline (RAS), and pramipexole (PRX) on synaptic plasticity in the frontal cortex of MPTP mice. **(A)** Experimental timeline. **(B)** The input–output curves of population spike amplitude (PSA) in the hippocampus–medial prefrontal cortex pathway of the control mice and the MPTP mice prior to drug treatment. The groups were control (*n* = 7), MPTP + saline (*n =* 7), MPTP + PRX (*n =* 5), MPTP + SEL (*n =* 6), and MPTP + RAS (*n =* 5). Values represent means ± SEM. 20% of maximum current: *F*_(4,25)_ = 0.867, *p* = 0.497; 40%: *F*_(4,25)_ = 0.354, *p* = 0.839; 60%: *F*_(4,25)_ = 1.132, *p* = 0.364; 80%: *F*_(4,25)_ = 1.419, *p* = 0.257. **(C)** Representative electrograms obtained before (black line) and after (red line) high-frequency stimulation (HFS). **(D)** Effects of SEL, RAS, and PRX on an HFS-induced sustainable increase in PSA. Left panel, changes in PSA; right panel, area under the curve (AUC) of PSA for 60 min after the first HFS. The groups were control (*n =* 7), MPTP + saline (*n =* 7), MPTP + PRX (*n =* 5), MPTP + SEL (*n =* 6), and MPTP + RAS (*n =* 5). Values represent means ± SEM. ^*^p < 0.05 versus the control group. ^#^*p* < 0.05 versus the MPTP + saline group. ^$^*p* < 0.05 versus the MPTP + RAS group (Tukey’s test), *F*_(4,25)_ = 7.922, *p* < 0.05. **(E)** Effects of SEL, RAS, and PRX on protein expression of CaMKIIα and CaMKIIβ, and their phosphorylation at threonine-286 in the frontal cortex. CaMKIIα (50 kDa), CaMKIIβ (60 kDa), and β-actin (42 kDa). The groups were control (*n =* 3), MPTP + saline (*n =* 3), MPTP + PRX (*n =* 3), MPTP + SEL (*n =* 3), and MPTP + RAS (*n =* 3). Values represent means ± standard deviation. ^*^p < 0.05 versus the control group. ^#^*p* < 0.05 versus the MPTP + saline group. ^$^*p* < 0.05 versus the MPTP + RAS group (Tukey’s test), CaMKIIα expression: *F*_(4,10)_ = 1.765, *p* = 0.212; CaMKIIα phosphorylation: *F*_(4,10)_ = 13.286, *p* < 0.05; CaMKIIβ expression: *F*_(4,10)_ = 1.860, *p* = 0.194; CaMKIIβ phosphorylation: *F*_(4, 10)_ = 2.165, *p* = 0.147. The numbers in parentheses indicate doses in mg/kg.

We aimed to clarify whether the decreased cortical monoamine content in MPTP mice and whether the enhancement of monoaminergic transmission by selegiline or pramipexole influence synaptic plasticity, because monoamine concentrations in the synaptic cleft modulate the threshold of synaptic plasticity (Goto et al., 2010). We performed electrophysiological recordings from the mPFC in MPTP mice after a single s.c. administration of selegiline (10 mg/kg, as an effective dose for depression-like behavior), rasagiline (1 mg/kg, based on the clinical dose ratio against selegiline), pramipexole (0.3 mg/kg, as an effective dose for depression-like behavior), or saline (Figure 4A). Among these groups, there were no differences in the baseline synaptic transmission rates assessed by the input–output curve for PSA in the mPFC (Figure 4B). As can be seen in Figures 4C,D, HFS in the hippocampus led to a sustained increase in PSA in the mPFC of control mice, indicating LTP induction. In contrast, corresponding LTP induction in the mPFC was not detected in MPTP mice after application of HFS (*p* = 0.005), suggesting that MPTP treatment induced impairment in cortical synaptic plasticity. Both selegiline (10 mg/kg) and pramipexole (0.3 mg/kg) significantly recovered the impaired LTP and the decreased AUC of PSA in the mPFC of MPTP mice up to the levels comparable to those in control mice (pramipexole-treated versus saline-treated MPTP mice, *p* = 0.017; selegiline-treated versus saline-treated MPTP mice, *p* = 0.002). However, rasagiline (1 mg/kg) failed to restore the impaired LTP in the mPFC of these mice.

Phosphorylation of CaMKIIα is crucial for LTP induction (Giese et al., 1998; Chang et al., 2017). In the present study, a significant decrease in CaMKIIα phosphorylation was detected in the frontal cortex of MPTP mice compared with that of control mice (*p* = 0.007). Selegiline (10 mg/kg) and pramipexole (0.3 mg/kg) normalized the MPTP-induced decrease in CaMKIIα phosphorylation (pramipexole-treated versus saline-treated MPTP mice, *p* = 0.003; selegiline-treated versus saline-treated MPTP mice, *p* = 0.002; Figure 4E), but rasagiline (1 mg/kg) did not. None of these drugs affected CaMKIIβ phosphorylation or the total expression of both CaMKII isoforms (Figure 4E). These results suggest that the MPTP-induced impairment of LTP is associated with reduced CaMKIIα activation, and that the restorative effects of selegiline and pramipexole on impaired LTP in MPTP mice are possibly mediated by CaMKIIα activation.

## Discussion

In the present study, we aimed to explore the antidepressant-like effects of propargyl MAOBIs, selegiline and rasagiline in MPTP mice, and to elucidate the mechanisms underlying these effects. Repeated injections of MPTP at 17.5 mg/kg led to some reduction in the number of nigrostriatal TH-positive neurons, causing depression- and anxiety-like behavior without any obvious induction of motor deficits. Some studies have demonstrated that rodent models of PD, including MPTP-treated mice and 6-hydroxydopamine-lesioned mice, show longer immobility time in the TST or the forced swim test, and more intensive motor impairment (Mori et al., 2005; Bonito-Oliva et al., 2014a). This phenotype in 17.5 mg/kg-MPTP mice is possibly in line with the clinical features of early PD, where NMS including depression and anxiety develop prior to the appearance of motor symptoms (Barone et al., 2009; Sauerbier et al., 2016). In the mice treated with 17.5 mg/kg MPTP, administration of pramipexole alleviated depression- and anxiety-like behavior. These effects of pramipexole are consistent with the results of previously reported studies in 6-hydroxydopamine-treated rodents (Berghauzen-Maciejewska et al., 2014; Bonito-Oliva et al., 2014a), as well as with the findings of clinical studies reporting that pramipexole is effective for treatment of depression in PD (Barone et al., 2010; Seppi et al., 2011). Thus, 17.5 mg/kg-MPTP mouse is a useful rodent model that exhibits a premotor phase, similar to a relative early stage of PD.

In contrast to medications for patients with MDD, even a single administration of typical antidepressants results in an amelioration of depression-like behavior in stressed mice (Steru et al., 1985; Bai et al., 2001; Yamada et al., 2013). In rodent models of PD, some antidepressants such as desipramine ameliorated depression-like behavior by single administration, but other antidepressants showed no significant efficacy for the depression-like behavior even after repeated administration (Chenu et al., 2007; Berghauzen-Maciejewska et al., 2014). Some clinical studies foresaw the possible effects of tricyclic antidepressants on depression in PD (Devos et al., 2008; Menza et al., 2009); however, a meta-analysis of randomized placebo-controlled trials showed no significant efficacy of antidepressants, including tricyclic antidepressants (Troeung et al., 2013). The findings of the meta-analysis suggested that the treatment of depression in PD may require some approaches different from the medications for MDD.

Selegiline is therapeutically effective for motor symptoms in patients with PD, and it alleviates clinical symptoms in patients with MDD (Mann et al., 1989). Rasagiline, another MAOBI, is more potent than selegiline in terms of MAO inhibition in a rodent brain *in vivo* (Youdim et al., 2001). In MPTP mice, the inhibitory effects of 10 mg/kg selegiline on MAO-A and MAO-B activities were comparable with those of 3 mg/kg rasagiline. Selegiline at 10 mg/kg suppressed depression-like behavior in MPTP mice, but rasagiline did not, even at a dose of 3 mg/kg that exerts relatively selective MAO-B inhibition. These results indicate that the antidepressant-like effects of selegiline in MPTP mice are independent of its MAO inhibitory capacity, consistent with the results of previous studies using naïve mice (Shimazu et al., 2005; Amiri et al., 2016; Ishikawa et al., 2019). Moreover, an L-Dopa equivalent dose (equivalent to 100 mg L-Dopa) of selegiline is 10-fold higher than that of rasagiline in patients with PD (Tomlinson et al., 2010). Compared with rasagiline at 1 mg/kg, since previous pharmacological studies (Abassi et al., 2004; Kasai et al., 2017) had evaluated based on this ratio, selegiline at 10 mg/kg ameliorated depression-like behavior and restored impaired synaptic plasticity in the mPFC of MPTP mice. The antidepressant-like effects of selegiline might be mediated through other mechanisms, because there are some pharmacological differences in DA reuptake inhibition (Lamensdorf et al., 1999) and preferentially inducible neurotrophic factors (Naoi et al., 2013) between these propargyl MAOBIs. A clinical trial reported that rasagiline had shown some improvement in PD depression (Barone et al., 2015); however, there have been no reports of a clinical trial evaluating the effect of selegiline on depression in PD as a primary endpoint. The results in this study revealed the antidepressant-like effects of selegiline in MPTP mice, indicating the possibility of its effectiveness on depression in patients with early PD.

At the highest dose of 10 mg/kg, selegiline normalized the elevated striatal DA turnover rate in MPTP mice. In these selegiline-treated MPTP mice, a positive correlation was observed between the striatal DA turnover rate and the immobility time in the TST. Notably, selegiline at 10 mg/kg markedly lowered the cortical DA turnover rate in MPTP mice, and there was a moderate correlation between the immobility time and cortical 5-HT and NA turnover rates. These results suggest that the antidepressant-like effect of selegiline in MPTP mice is partially concurred by the normalization of nigrostriatal DAergic system dysfunction and by the enhancement of cortical monoaminergic systems, because a monoamine turnover rate is used as an indirect indicator of presynaptic monoaminergic release (Schulte-Herbrüggen et al., 2012). Likewise, a single administration of pramipexole to MPTP mice improved depression-like behavior and lowered the elevated striatal DA turnover rate. In the forced swim test using naïve mice, a single administration of pramipexole exerted antidepressant-like effects that were mediated by activation of D_2_ receptors (Siuciak and Fujiwara, 2004; Ostadhadi et al., 2016), and led to a reduction in the striatal DA turnover rate, indicating decreases in released DA levels (Schulte-Herbrüggen et al., 2012). Antidepressant-like effects of pramipexole in MPTP mice may also be attributable to the normalization of nigrostriatal DAergic system dysfunction. The mesocortical DAergic pathway as well as the nigrostriatal pathway has been reported to be sensitive to MPTP treatment (Rousselet et al., 2003). In the present study, administration of MPTP at a dose of 17.5 mg/kg caused a significant reduction in DA content of the cerebral cortex, whereas no significant reduction was observed in the number of TH-positive cells in the ventral tegmental area of MPTP mice (data not shown). These findings are in agreement with those of previous reports (Rousselet et al., 2003; Neirinckx et al., 2013). Thus, a reduction in DA content in the frontal cortex of the present MPTP mice might result from the dysfunction of nerve terminals rather than degradation of cell bodies in the ventral tegmental area–PFC DAergic pathway. Moreover, the hippocampus–PFC pathway is glutamatergic (Parent et al., 2010) and assumed to be associated with the pathology of psychiatric symptoms in PD (Takahashi et al., 2008). In the present study, HFS in the hippocampus did not induce LTP in the mPFC of MPTP mice. To our knowledge, there are no *in vivo* studies demonstrating the impairment of LTP in the hippocampus–mPFC pathway in PD animal models, although several studies have shown the impairment of HFS-induced hippocampal LTP, which may lead to cognitive dysfunction in PD rodent models (Costa et al., 2012; Diógenes et al., 2012; Moriguchi et al., 2012; Bonito-Oliva et al., 2014b; Castro-Hernández et al., 2017). Similar to patients with MDD (Normann et al., 2007; Kuhn et al., 2016), depressed patients with PD may have impairment in cortical LTP-like plasticity. Furthermore, both selegiline and pramipexole restored LTP impairment in the mPFC of MPTP mice at the ameliorating doses for depression-like behavior. The impairment in HFS-induced LTP in the hippocampus–PFC pathway can be reversed by some antidepressants in rats exposed to stress (Rocher et al., 2004), although, to our knowledge, there are no previous reports regarding correlation between antidepressants’ efficacies and their restorative potential for LTP impairment in the mPFC of MPTP mice. The mechanisms underlying the antidepressant-like effects of selegiline and pramipexole in MPTP mice may be attributable to the restoration of LTP impairment in the hippocampus–mPFC pathway. In addition, our recent study demonstrated the ameliorating effect of selegiline on depression-like behavior in rodents subjected to the TST and the forced swim test, and its preventative effect on hippocampal CA1 LTP impairment induced by low-frequency stimulation in naïve rats (Ishikawa et al., 2019). Although there are several reports about the association of physical and functional changes in hippocampus, as a region for emotional perception, with depression severity of patients with PD (van Mierlo et al., 2015; Györfi et al., 2017; Gou et al., 2018), there have been no reports showing the relationship between hippocampal LTP impairment and depression-like behavior in PD animal models. Further studies are required to clarify whether MPTP mice showing depression-like behavior have any hippocampal LTP impairment, and whether selegiline has the potential to restore MPTP-induced impairment in terms of synaptic plasticity in the hippocampus.

Ca^2+^/calmodulin-dependent autophosphorylation at threonine-286 of CaMKIIα induces its Ca^2+^-independent activity, namely “autonomy” (Lisman et al., 2012). In addition, phosphorylation at threonine-286 of CaMKIIα is crucial for LTP induction and learning as reported in the study with mice having a point mutation at threonine-286 of CaMKIIα and exhibiting hippocampal LTP impairment and spatial learning deficit (Giese et al., 1998; Chang et al., 2017). A reduction in CaMKIIα phosphorylation gives rise to hippocampal LTP impairment in MPTP mice (Moriguchi et al., 2012). Phosphorylation on threonine-286 of CaMKII is enhanced by activation of D_1_ receptors (Anderson et al., 2008) or D_1_–D_2_ heteromeric receptor complexes (Rashid et al., 2007). Depression-like behavior in MPTP mice has been reported to result from the impairment of D_1_ receptor-mediated neurogenesis in the hippocampus (Zhang et al., 2016). In the present study, selegiline and pramipexole normalized the reduced phosphorylation of CaMKIIα in the frontal cortex of MPTP mice. Therefore, the restorative effects of selegiline on impaired synaptic plasticity and reduced CaMKIIα activation in the mPFC of MPTP mice may be induced through activation of D_1_ receptors, followed by an increase in CaMKIIα phosphorylation. This is consistent with our previous study, in which selegiline exerted antidepressant-like effects via activation of D_1_ receptors in naïve mice under the forced swim test (Shimazu et al., 2005). Likewise, CaMKII has been demonstrated to interact with D_2_ and D_3_ receptors (Liu et al., 2009; Zhang et al., 2014). Activation of D_3_ receptor, a member of D_2_-like receptor family, increased LTP in a mouse hippocampal slice (Swant and Wagner, 2006), and the D_3_-preferring agonist, pramipexole, restored LTP impairment in a hippocampal slice from MPTP mice (Castro-Hernández et al., 2017). Thus, in the present study, the restorative effects of pramipexole on the impaired LTP induction and on the reduced activation of CaMKIIα in the mPFC of MPTP mice may be mediated through activation of D_3_ receptors.

In contrast to depression-like behavior, a single administration of either selegiline or rasagiline at any dose did not improve anxiety-like behavior in MPTP mice. Although we did not investigate whether repeated exposure to either MAOBI leads to anxiolytic effects, the results herein are in line with the findings of randomized, placebo-controlled trials in which rasagiline was less effective in treating anxiety in PD (Hanagasi et al., 2011; Smith et al., 2015). Selegiline and rasagiline may thus be less effective for treating anxiety in PD at the doses therapeutically effective for treating motor symptoms. Anxiety and depression frequently coexist in patients with PD, but these two symptoms are differentially associated with demographic, clinical, or therapeutic features (Nègre-Pagès et al., 2010; Wee et al., 2016). Anxiety-like behavior in MPTP mice may not share a common pathology with that of depression-like behavior. In the present study, MPTP mice showed anxiety-like behavior and a decrease in the distance traveled in the EPM (Supplementary Figure 2), the environment of which is more stressful than that in a cage while measuring spontaneous locomotor activities. Treatment with pramipexole relieved anxiety-like behavior without recovery of decrease in the distance traveled in the EPM (Supplementary Figure 2). Previous studies have demonstrated that the state of anxiety in PD can interfere with information processing, resulting in the exacerbation of gait impairment in stressful situations, and that DAergic treatment might improve freezing of gait due to its anxiolytic effects (Ehgoetz Martens et al., 2014, 2015). However, further investigation is required to clarify whether DAergic treatments have the potential to improve freezing of gait through its anxiolytic effects under highly stressful conditions.

The present study has some limitations: (1) the antidepressant-like activity of selegiline was evaluated based on a single (not chronic) administration treatment design and (2) degeneration of the nigrostriatal DAergic neurons was evaluated only by TH immunohistochemical staining. We cannot exclude the possibility that the effective dose (10 mg/kg) of selegiline in MPTP mice is higher than the therapeutic doses for patients with PD (5–10 mg/day, oral) because of a single administration design and pathological differences between acute MPTP mice and PD patients. Therefore, further analyses using rodent models of chronic PD (e.g., the MPTP/probenecid chronic model) may clarify the effects of chronic administration of selegiline on NMS-like behavior in PD and on the expression levels of other proteins associated with synaptic plasticity, such as brain derived neurotrophic factors. Regarding the second limitation, a reduction in expression of TH protein and mRNA in the surviving DAergic neurons was observed in MPTP-treated mice (Petzinger et al., 2007) and patients with PD (Javoy-Agid et al., 1990). However, it has been reported that MPTP treatment regimen (16.4 mg free base/kg, at 2-h intervals, similar to the regimen in this paper) induced a comparable reduction in TH-positive cells to that in Nissl-positive cells in the substantia nigra at 1 and 9 days post-injection (Liu et al., 2016). Thus, reduction in the striatal TH-positive fiber density and the number of TH-positive nigral cells in MPTP mice observed in the present study may not reflect the actual loss of DAergic fiber and neurons.

## Conclusion

In conclusion, the present study demonstrates that the features of 17.5 mg/kg-MPTP mouse simulate a part of those in premotor phase of PD by exhibiting depression-like behavior, striatal and cortical monoaminergic dysfunction, and LTP impairment in the mPFC. A single administration of selegiline or pramipexole, but not rasagiline, ameliorates the depression-like behavior. In addition, selegiline and pramipexole, but not rasagiline, restored both LTP impairment in the mPFC and the reduced CaMKIIα activation in the frontal cortex of MPTP mice. These results suggest that the antidepressant-like effect of selegiline is partially attributable to the improvement in nigrostriatal and cortical DAergic dysfunction, and to the restoration of impaired synaptic plasticity in the mPFC through CaMKIIα phosphorylation. Our findings highlight the potential efficacy of selegiline as a monotherapeutic anti-PD agent for the treatment of both motor dysfunctions and depression.

## Data Availability

The datasets generated for this study are available on request to the corresponding author.

## Ethics Statement

All animal procedures were carried out in accordance with the applicable international, national, and institutional guidelines for the care and use of animals. All experiments were approved by the Committee of Fujimoto Pharmaceutical Corporation on Animal Experimentation (the approval number: AC-F-2666).

## Author Contributions

KT and MO conceived and designed the research. MO and JS performed the experiments and analyzed the data. MO wrote the initial draft of the manuscript. KT and SM revised the manuscript. All authors reviewed the final manuscript and approved its publication.

## Conflict of Interest Statement

All authors are employees of Fujimoto Pharmaceutical Corporation. This study was funded by the Fujimoto Pharmaceutical Corporation, to which all authors affiliate. This research did not receive any specific grant from funding agencies in the public or not-for-profit sectors.
