# Supplementary material for: Selegiline Recovers Synaptic Plasticity in the Medial Prefrontal Cortex and Improves Corresponding Depression-Like Behavior in a Mouse Model of Parkinson’s Disease
Source: Front Behav Neurosci. 2019 Aug 2;13:176. doi: 10.3389/fnbeh.2019.00176 (PMC6688712; doi:10.3389/fnbeh.2019.00176)
Supplement: Supplementary file 1 [file Table_1.doc]

***Supplementary Material***

**Selegiline Recovers Synaptic Plasticity in the Medial Prefrontal Cortex and Improves Corresponding Depression-Like Behavior in a Mouse Model of Parkinson's Disease**

Motoki Okano^1^, Kazue Takahata^1*^, Junya Sugimoto^1^ and Shizuko Muraoka^1^

^1^Department of Scientific Research, Fujimoto Pharmaceutical Corporation, Osaka, Japan

*Correspondence:

Email: [k-takahata@fujimoto-pharm.co.jp](mailto:k-takahata@fujimoto-pharm.co.jp)

**
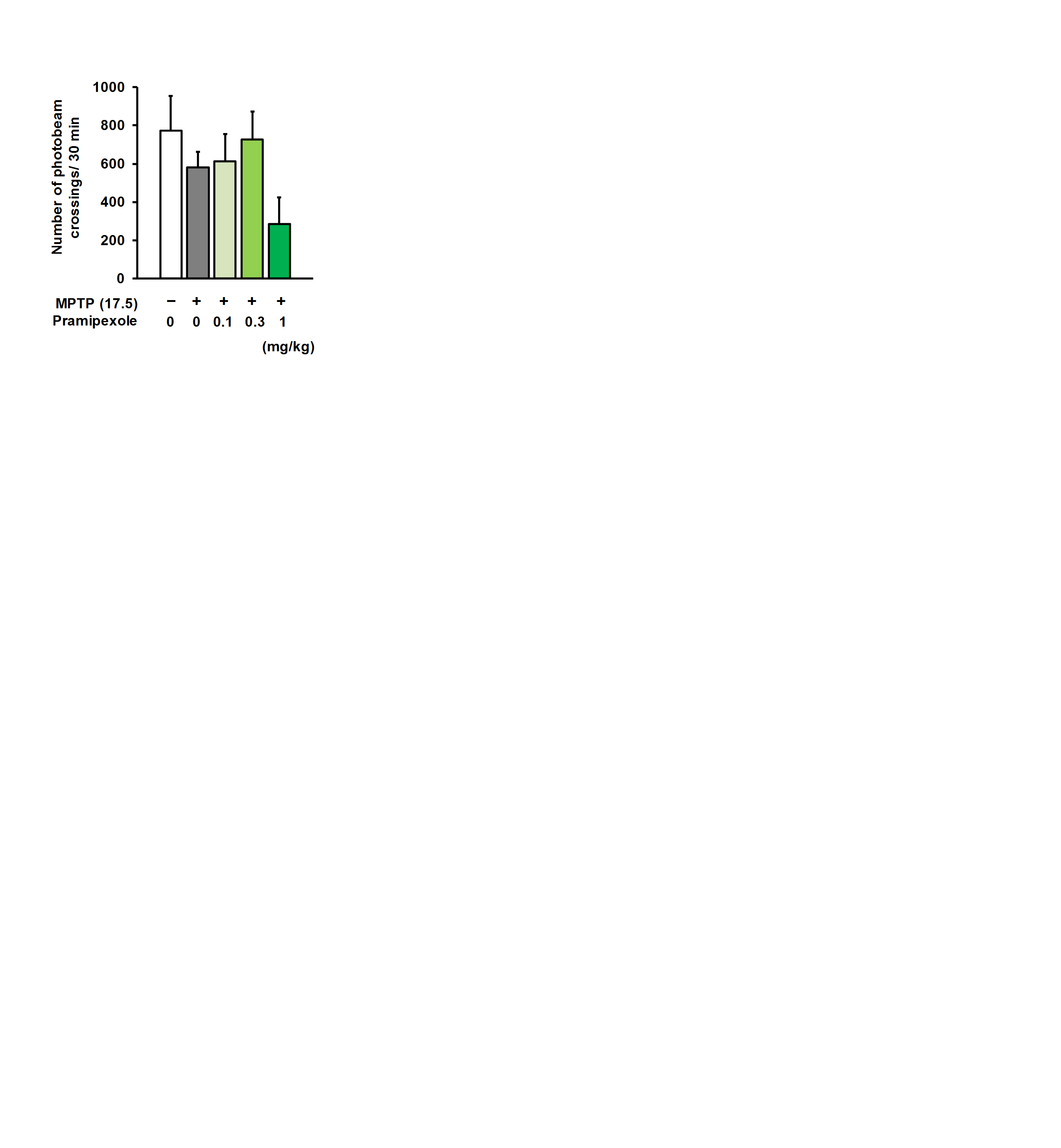
**

**Supplementary Figure 1**

**Effect of pramipexole (PRX) on spontaneous locomotor activity in MPTP mice.**

The groups were control (n = 3), MPTP (n = 4), MPTP + 0.1 mg/kg PRX (n = 3), MPTP + 0.3 mg/kg PRX (n = 4), and MPTP + 1 mg/kg PRX (n = 3). Values represent means ± SD. *F* _(4,12)_ = 1.509, *p* = 0.261.


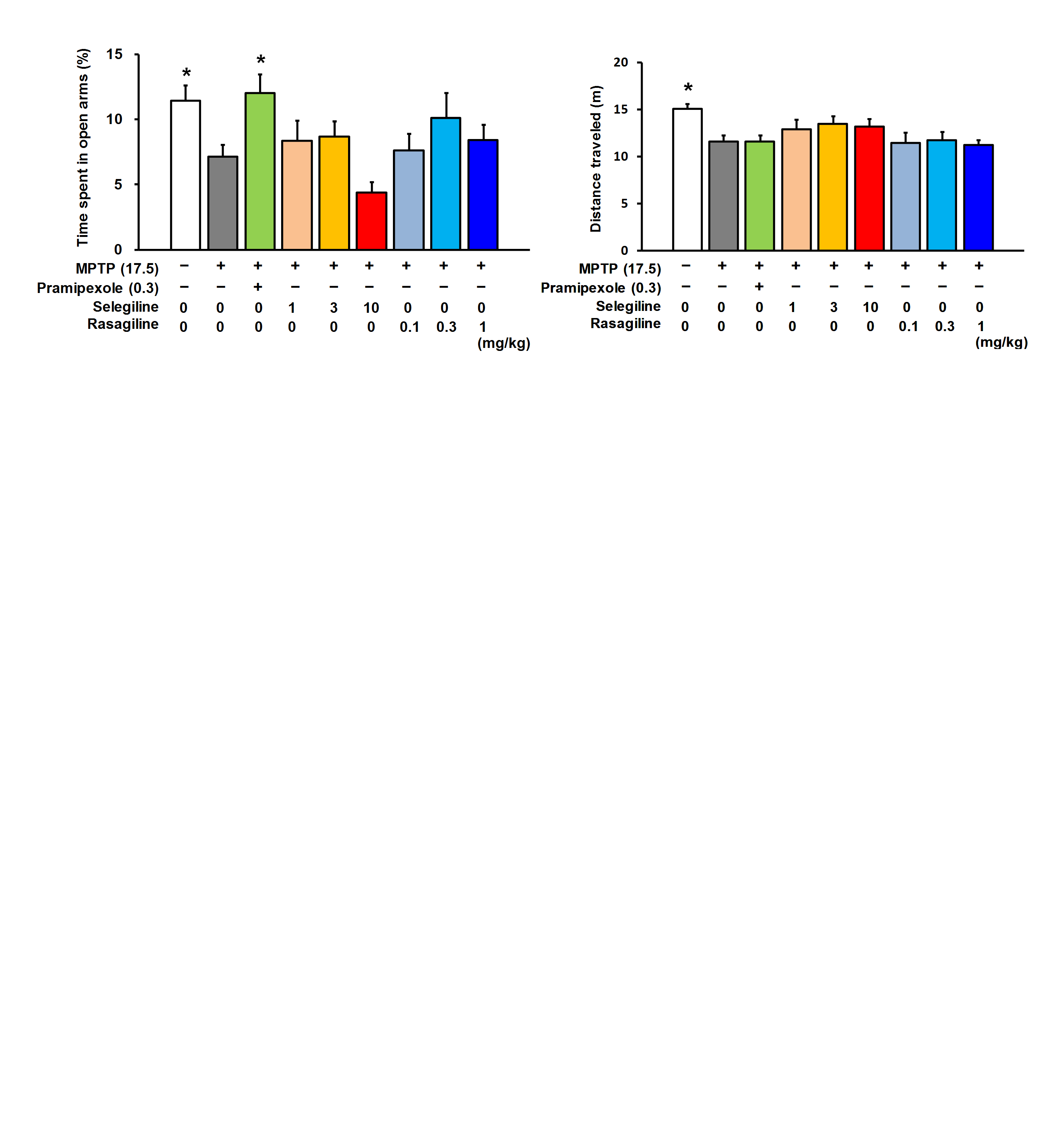


**Supplementary Figure 2**

**Effects of monoamine oxidase B inhibitors, selegiline (SEL) and rasagiline (RAS), and pramipexole (PRX) on anxiety-like behavior in the elevated plus maze test in MPTP mice**

MPTP mice showed a reduction in time length spent in the open arms (left panel) and a decrease in the distance traveled (right panel) in the elevated plus maze test. The groups were control (*n =* 37), MPTP + saline (*n =* 36), MPTP + PRX (*n =* 22), MPTP + 1 mg/kg SEL (*n =* 14), MPTP + 3 mg/kg SEL (*n =* 13), MPTP + 10 mg/kg SEL (*n =* 14), MPTP + 0.1 mg/kg RAS (*n =* 14), MPTP + 0.3 mg/kg RAS (*n =* 13), and MPTP + 1 mg/kg RAS (*n =* 14). Values represent means ± SEM. **p* < 0.05 versus the MPTP + saline group (Dunnett’s test), time spent in open arms: *F* _(8,168)_ = 3.207, *p* < 0.05; distance traveled: *F* _(8,168)_ = 3.823, *p* < 0.05.


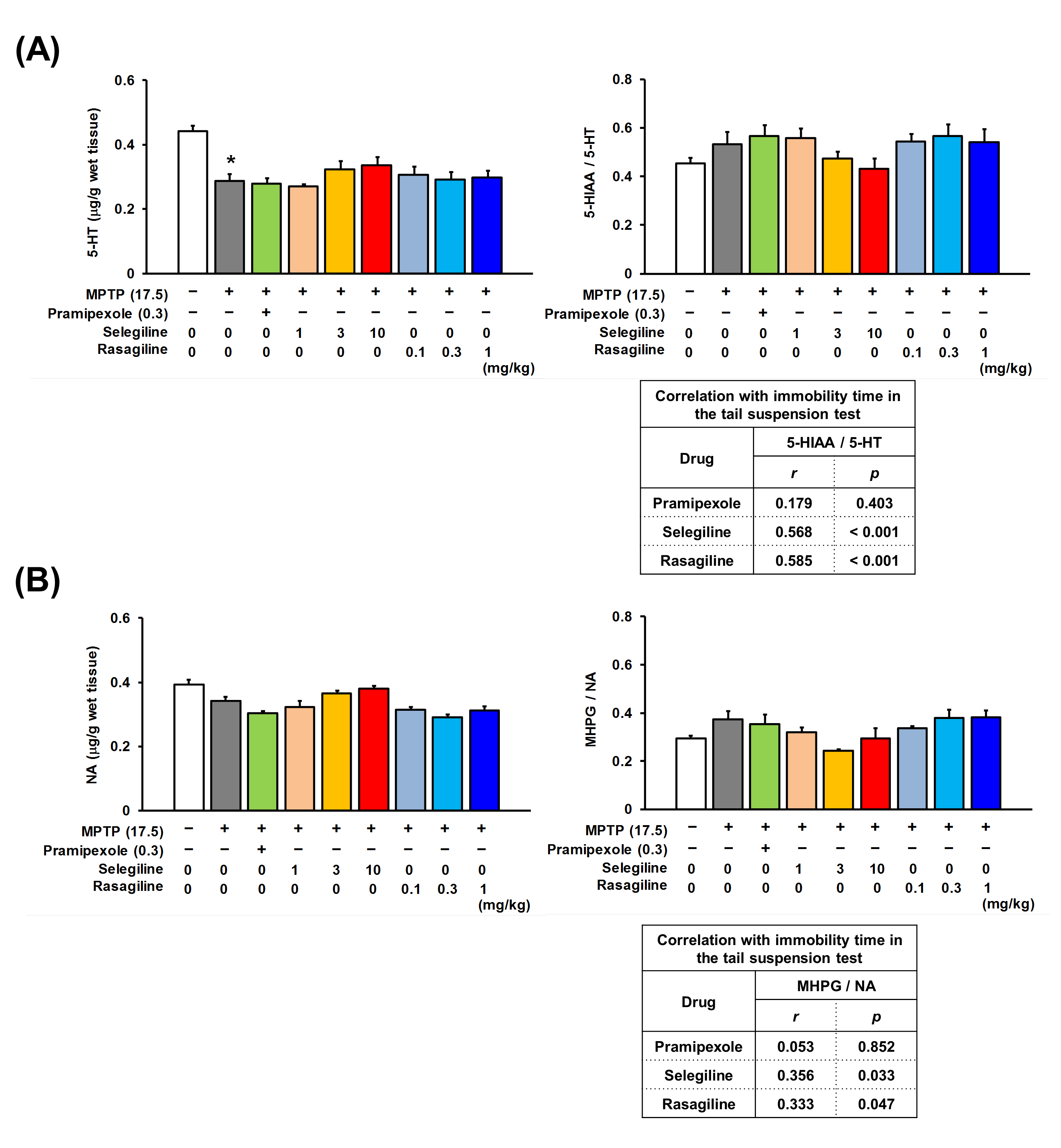


**Supplementary Figure 3**

**Effects of selegiline (SEL), rasagiline (RAS), and pramipexole (PRX) on serotonin (5-HT) and norepinephrine (NA) content and their turnover rates in the cortex of MPTP mice**

(A–B) Effects of SEL, RAS, and PRX on 5-HT (A) and NA (B) content and their turnover rates in the cortex. The groups were control (*n =* 8), MPTP + saline (*n =* 6), MPTP + PRX (*n =* 8), MPTP + 1 mg/kg SEL (*n =* 7), MPTP + 3 mg/kg SEL (*n =* 7), MPTP + 10 mg/kg SEL (*n =* 8), MPTP + 0.1 mg/kg RAS (*n =* 7), MPTP + 0.3 mg/kg RAS (*n =* 7), and MPTP + 1 mg/kg RAS (*n =* 8). Values represent means ± SEM. **p* < 0.05 versus the control group, 5-HT content: *F* _(8,57)_ = 5.746, *p* < 0.05, 5-hydroxyindoleacetic acid (5-HIAA)/5-HT: *F* _(8,57)_ = 1.518, *p* = 0.171; NA content: *F* _(8,57)_ = 7.552, *p* < 0.05, 3-methoxy-4-hydroxyphenylglycol (MHPG)/NA: *F* _(8,57)_ = 2.336, *p* < 0.05.
